# Risk Factors for Metastatic Infectious Complications in Patients with Catheter-Related Bloodstream Infection

**DOI:** 10.3390/antibiotics15101015

**Published:** 2026-10-13

**Authors:** Audrey Lanyan, Nicolas Fourré, Virgile Zimmermann, Laurence Senn, Benoit Guery, Matthaios Papadimitriou-Olivgeris

**Affiliations:** 1 Infectious Diseases Service, Lausanne University Hospital and University of Lausanne, 1011 Lausanne, Switzerlandnicolas.fourre@ehnv.ch (N.F.); virgile.zimmermann@chuv.ch (V.Z.); laurence.senn@chuv.ch (L.S.); benoit.guery@chuv.ch (B.G.); 2 Infectious Diseases Service, Hospital of Valais and Institut Central des Hôpitaux, 1951 Sion, Switzerland

**Keywords:** catheter-related bacteremia, bloodstream infection, infective endocarditis, bone and joint infections, *Staphylococcus aureus*, spondylodiscitis, vascular graft infection, embolic events, septic deep vein thrombosis, persistent bacteremia

## Abstract

**Background/Objectives:** Catheter-related bloodstream infections (CRBSIs) may lead to metastatic complications. However, risk factors for such complications remain insufficiently characterized. This study aims to identify risk factors associated with metastatic infectious complications in patients with CRBSI. **Methods:** This monocentric cohort study included adult patients with CRBSI due to Gram-positive cocci or *Candida* spp. (2015–2024). The primary endpoint was the occurrence of metastatic complications, including infective endocarditis, embolic events, bone and joint infections, and vascular graft infections. **Results:** A total of 1023 CRBSI episodes were included. *Staphylococcus aureus* was the most common pathogen (331 episodes; 32%), followed by coagulase-negative staphylococci (307; 30%), enterococci (175; 17%), *Candida* spp. (171; 17%), and streptococci (98; 10%). Metastatic complications occurred in 93 episodes (9%). In multivariable logistic regression, female sex (aOR 1.61; 95% CI 1.02–2.56), *S. aureus* infection (aOR 1.62; 95% CI 1.01–2.61), community-onset infection (aOR 2.23; 95% CI 1.26–3.95), septic deep vein thrombosis (aOR 2.19; 95% CI 1.04–4.58), and persistent bacteremia ≥ 48 h (aOR 4.54; 95% CI 2.78–7.44) were independently associated with metastatic complications. In the *S. aureus* subgroup, only persistent bacteremia remained significant (aOR 6.28; 95% 3.12–12.6). **Conclusions:** Metastatic complications occurred in approximately 9% of CRBSI episodes but were strongly associated with persistent bacteremia, particularly in *S. aureus* infections. Persistence of bloodstream infection and community-onset presentation should prompt evaluation for metastatic foci.

## 1. Introduction

The use of intravascular catheters has increased substantially over recent decades, with a growing proportion of patients managed in the ambulatory setting [1,2,3]. This trend is driven by increasing patient complexity and the expanding use of prolonged therapies, including chemotherapy, long-term antimicrobial treatment, and parenteral nutrition [1,2,3]. Despite improvements in catheter care and infection prevention strategies, catheter-related bloodstream infections (CRBSIs) remain a major source of healthcare-associated infections, accounting for up to 10–20% of cases depending on the clinical setting [4,5,6,7,8,9]. Gram-positive cocci, particularly coagulase-negative staphylococci and *Staphylococcus aureus*, are the most frequently implicated pathogens [8,9].

CRBSI can be complicated by metastatic infections, including embolic events (notably pulmonary septic emboli), infective endocarditis, bone and joint infections, and vascular graft or endograft infections [10,11,12,13,14,15,16]. Although relatively uncommon, these complications occur in approximately 4–19% of cases and are associated with substantial morbidity and mortality [10,11,12,13,14]. Early identification is therefore critical, as it directly influences diagnostic and therapeutic management, including the use of targeted imaging, prolonged antimicrobial therapy, and, in some cases, surgical intervention [17,18].

Several risk factors for metastatic infectious complications have been previously identified. These include pathogen-related factors, particularly *S. aureus* infection, as well as host-related factors such as hemodialysis, immunosuppression, and the presence of intracardiac, endovascular, or osteoarticular prosthetic material [10,11,13]. However, data are mainly derived from small studies, particularly those focused on *S. aureus* alone; they remain limited and heterogeneous, and risk stratification in patients with CRBSI is not well standardized.

The aim of this study was to identify risk factors associated with metastatic infectious complications in a large cohort of patients with CRBSI.

## 2. Results

**Figure 1:**
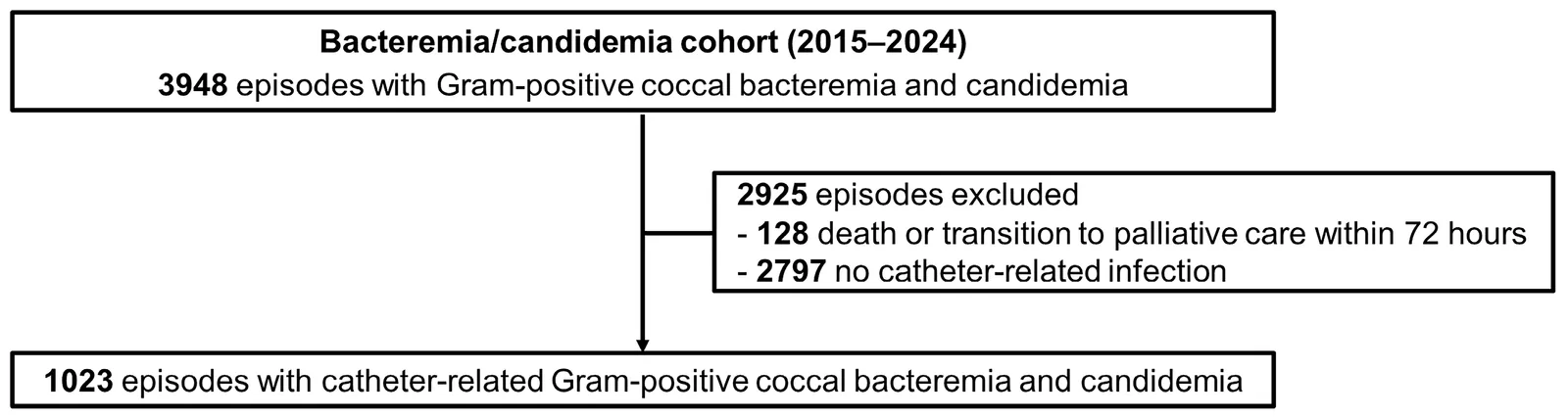
Flowchart of included episodes.

Among the 3948 episodes of bacteremia and candidemia identified in the cohort, 1023 CRBSI episodes caused by Gram-positive cocci and *Candida* spp. were included in the analysis (Figure 1). *S. aureus* was the most common pathogen (331 episodes; 32%), followed by coagulase-negative staphylococci (307; 30%), enterococci (175; 17%), *Candida* spp. (171; 17%), and streptococci (98; 10%). Polymicrobial bacteremia/candidemia was identified in 156 (15%) episodes.

CRBSI involved a peripheral venous catheter in 260 episodes (25%) and a central venous catheter in 763 episodes (75%); among the latter, 435 (57%) were short-term central venous catheters, 146 (19%) peripherally inserted central catheters, 143 (19%) totally implantable venous access devices, and 39 (5%) permanent nephrological catheters.

ID consultation was obtained in 953 episodes (93%), and follow-up blood cultures until documented clearance were performed in 996 (98%). Transthoracic echocardiography, transesophageal echocardiography, [^18^F]fluorodeoxyglucose positron emission tomography/CT ([^18^F]FDG-PET/CT), and cardiac CT were performed in 563 (59%), 158 (15%), 83 (8%), and 14 (1%) episodes, respectively. Thoracoabdominal CT was performed in 494 episodes (48%), venous duplex ultrasonography in 474 (46%), spinal MRI in 45 (4%), and cerebral imaging in 204 (20%).

Overall, 93 episodes (9%) were complicated by metastatic infection. Among these, 63 (68%) had embolic events, 30 (32%) were diagnosed with infective endocarditis, 10 (11%) had bone and joint infections, and 3 (3%) had vascular graft or endograft infections; 12 (13%) episodes presented with two metastatic infectious complications.

Recurrence of bacteremia/candidemia within 90 days of the initial episode was observed in 22 (2%) episodes: 21 of 930 (2%) episodes without metastatic infectious complications and 1 of 93 (1%) episodes with metastatic infectious complication (Table S1). None of the recurrent episodes of bacteremia/candidemia were associated with metastatic complications.

**Figure 2:**
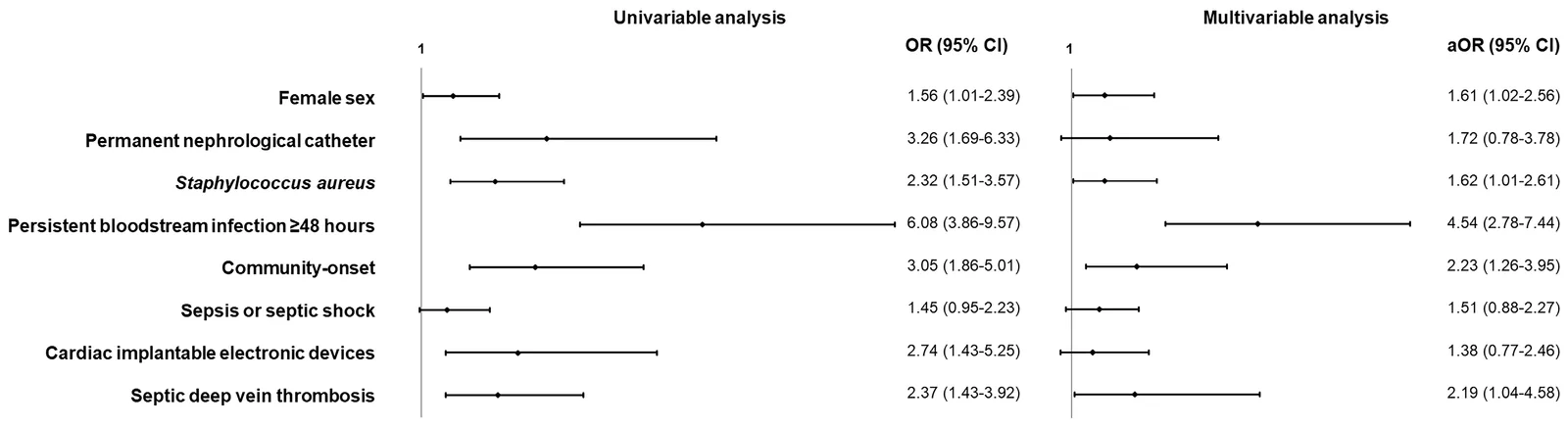
Forest plot of univariable and multivariable logistic regression of metastatic infectious complications among 1023 episodes with catheter-related bacteremia. aOR: adjusted odds ratio; CI: confidence interval.

The comparison between episodes with and without metastatic infectious complications among the 1023 CRBSI episodes is presented in Table 1. In multivariable logistic regression analysis, female sex (aOR 1.61; 95% CI 1.02–2.56), *S. aureus* CRBSI (aOR 1.62; 95% CI 1.01–2.61), persistent bloodstream infection ≥48 h (aOR 4.54; 95% CI 2.78–7.44), community-onset infection (aOR 2.23; 95% CI 1.26–3.95), and the presence of septic deep vein thrombosis (aOR 2.19; 95% CI 1.04–4.58) were independently associated with metastatic infectious complications (Figure 2, Table S2).

Among the 331 episodes of *S. aureus* CRBSI, 47 (14%) had metastatic infectious complications. Of these, 31 (65%) had embolic events, 19 (40%) had infective endocarditis, 4 (9%) had bone and joint infections, and 2 (4%) had vascular graft infections; 9 (19%) episodes presented with two metastatic infectious complications.

**Figure 3:**
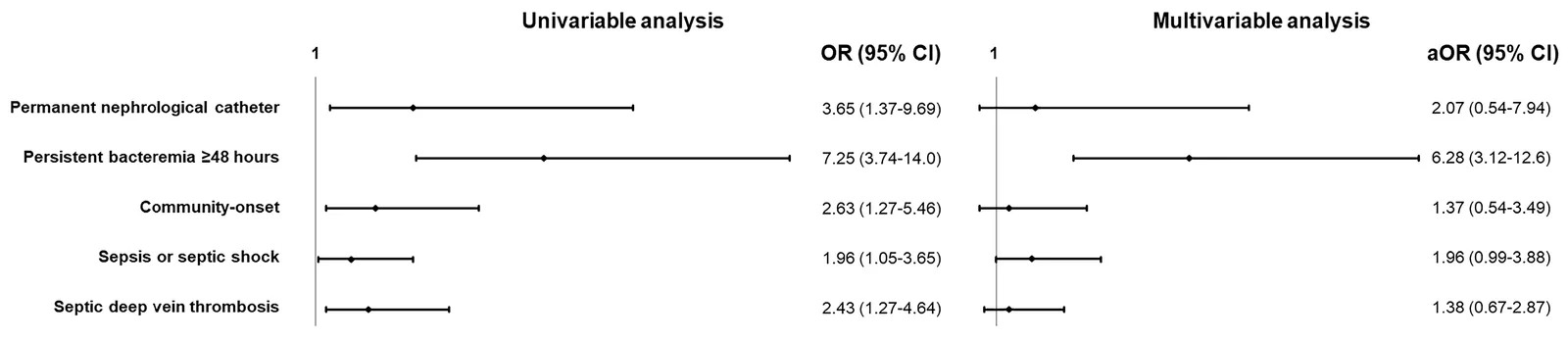
Forest plot of univariable and multivariable logistic regression of metastatic infectious complications among 331 episodes with catheter-related *S. aureus* bacteremia. aOR: adjusted odds ratio; CI: confidence interval.

The comparison of episodes with and without metastatic infectious complications among *S. aureus* CRBSI is shown in Table 2. In multivariable analysis, only persistent bacteremia ≥ 48 h was independently associated with metastatic infectious complications (aOR 6.28; 95% CI 3.12–12.6) (Figure 3, Table S3).

## 3. Discussion

In our cohort, 9% of episodes with CRBSI caused by Gram-positive cocci and *Candida* spp. were complicated by metastatic infection, the occurrence of which was associated with several infection-, pathogen-, and host-related factors.

The rate of metastatic infectious complications among CRBSI was comparable to that previously reported [11,12,13,14,19]. This rate varies according to the causative pathogen, with *S. aureus* CRBSI consistently shown to carry the highest risk of such complications, not only among CRBSIs but also in bacteremia overall [12,19]. The high pathogenicity of *S. aureus* is driven by multiple virulence factors, including adhesins, toxins, and coagulases, which promote host tissue adherence, immune evasion, activation of the coagulation cascade, abscess formation, persistence in blood and tissues, and hematogenous dissemination to distant sites such as heart valves, bones, and joints [20,21,22,23].

Persistent positive follow-up blood cultures were also associated with metastatic infectious complications, a feature particularly prominent in *S. aureus* bacteremia [10,19,24,25]. Persistent bacteremia reflects failure of infection clearance and sustained intravascular microbial burden, thereby increasing the likelihood of hematogenous seeding. Such failure to clear bacteremia has been associated with inadequate antimicrobial therapy or delayed source control when indicated [11,16,19]. In the specific context of CRBSI, persistent positive follow-up blood cultures are a recognized hallmark of septic deep vein thrombosis, which in the present study was also associated with metastatic infectious complications [25,26].

Although the majority of CRBSI episodes occurred during hospitalization, to the best of our knowledge this is the first study to identify community-onset infection being independently associated with metastatic infectious complications [1,8]. This may be explained by a longer interval between symptom onset and microbiological diagnosis, allowing bacteremia to persist before appropriate antimicrobial therapy and source control are initiated, thereby facilitating the development of secondary infectious foci [27,28]. Community-onset infection has consistently been identified as a risk factor for metastatic complications, including infective endocarditis and bone and joint infections, in patients with bacteremia due to Gram-positive cocci [29,30].

Regarding catheter type, the incidence of metastatic infectious complications was higher among episodes involving permanent nephrological catheters, in line with previous reports [11,13]. This association may be explained by prolonged catheter dwell time, which promotes biofilm formation on the catheter surface and may contribute to persistent infection and subsequent hematogenous dissemination.

An unexpected finding was the higher rate of metastatic infectious complications among female patients compared to male patients. However, the association should be interpreted with caution, since no biological or clinical explanation is readily apparent. Our findings contrast with the previous literature, in which male sex has been identified as a risk factor for CRBSI [31], although this association has not been consistently observed [32]. Conversely, female sex has in some studies been associated with worse outcomes [33]. Notably, the available literature does not specifically address sex-related differences in the risk of metastatic complications arising from CRBSI. In the present study, this difference could not be explained by other measured factors, as no significant differences between male and female patients were observed with regard to catheter type, causative pathogens, presence of intravascular or osteoarticular prosthetic material, persistent bacteremia/candidemia, or community-onset infection.

This study has several limitations. First, it is a single-center retrospective study conducted in a university hospital with standardized evaluation by ID specialists and broad access to imaging modalities, which may limit generalizability to other settings. However, to the best of our knowledge, it represents the largest study to date evaluating the risk of metastatic complications in patients with CRBSI. Second, the definition of CRBSI deviates from that proposed by the Infectious Diseases Society of America [18]. In routine clinical practice, not all catheters are removed for tip culture, and when performed, cultures may occur after several days of antimicrobial therapy, reducing diagnostic yield. In addition, paired peripheral and catheter blood cultures required to calculate differential time to positivity are not systematically obtained. To mitigate potential misclassification, the diagnosis of CRBSI was adjudicated by the treating ID consultant, incorporating clinical criteria such as local signs of infection at the catheter insertion site (inflammation and/or purulent discharge) and the absence of an alternative plausible source of infection. Third, not all pathogens were included. Restricting inclusion to CRBSI due to Gram-positive cocci and *Candida* spp. was a pragmatic choice made to keep the study feasible: during the study period (2015–2024) at CHUV, *Escherichia coli* alone accounted for 3114 episodes of bacteremia, compared with 1401 episodes of *S. aureus* bacteremia, and extending data collection to Gram-negative organisms would have required substantially greater resources. The exclusion of pathogens less frequently associated with metastatic complications, such as Enterobacteriaceae [11], may have led to an overestimation of the overall risk. Fourth, the duration of catheter placement, a well-recognized risk factor for CRBSI [4,5,6,7,8,9], was not available for the majority of cases and could therefore not be included in the analysis. Fifth, there may be a detection or ascertainment bias related to the investigation of metastatic infectious complications, as diagnostic workup was directed by infectious diseases specialists, and imaging studies, including TEE, [^18^F]FDG-PET/CT, thoracoabdominal CT, cerebral imaging, and venous Doppler ultrasound, were ordered based on the presence of symptoms and/or predefined risk factors. Consequently, patients with a clinical suspicion of metastatic infection were more likely to undergo a more extensive and targeted imaging workup, which could in principle have increased the detection rate of metastatic complications in this group independently of their true underlying risk. This risk-adapted approach to imaging is consistent with recent guidelines for the management of *S. aureus* bacteremia, which recommend tailoring the intensity of imaging to the estimated risk of metastatic complications rather than performing whole-body imaging systematically in all patients [34]. Under these recommendations, extensive imaging, including whole-body modalities such as [^18^F]FDG-PET/CT, is reserved for patients at increased risk of metastatic infection with bacteremia of unknown origin after initial clinical assessment, rather than being applied uniformly. Several elements, however, suggest that detection or ascertainment bias is unlikely to have substantially altered our findings. A substantial proportion of episodes without metastatic infectious complications also underwent imaging investigations, indicating that diagnostic evaluation was not restricted to patients with an a priori high suspicion of metastatic disease. Second, episodes without metastatic infectious complications received a median antimicrobial treatment duration of 14 days, which would likely have been insufficient to treat an unrecognized metastatic focus, and none developed recurrent bacteremia/candidemia associated with metastatic complications during follow-up, arguing against a clinically significant number of missed diagnoses in this group. Finally, septic deep vein thrombosis was not considered a metastatic complication in this study, in contrast to some previous reports [11], as we considered it a contiguous complication directly related to the catheter.

## 4. Materials and Methods

This retrospective study was conducted at the CHUV, Switzerland, and incorporated episodes from the bacteremia/candidemia cohort, including episodes from January 2015 to December 2024. The study was approved by the Ethics Committee of the Canton of Vaud (CER-VD 2021-02516).

Inclusion criteria were adult patients (≥18 years) with CRBSI due to staphylococci, streptococci, enterococci, or *Candida* spp., and no recorded objection to the use of their data. Exclusion criterion comprised patients who died or transitioned to palliative care within 72 h of diagnosis.

Demographic, clinical, imaging, surgical, and microbiological data were manually extracted from electronic health records by two internal medicine consultants, one internal medicine resident, and one infectious diseases (ID) consultant. All data were subsequently reviewed and validated by an ID consultant.

Blood cultures were incubated using the BacT/ALERT System (bioMerieux, Marcy l’Etoile, France) for 72 h. Species identification was performed using matrix-assisted laser desorption–ionization time of flight mass spectrometry (MALDI-TOF MS; Bruker Daltonics, Bremen, Germany).

Definitions of bacteremia and candidemia according to clinical context are detailed in Table S4. An episode was classified as CRBSI if at least one of the following criteria was met: (i) a positive catheter tip culture yielding the same microorganism as identified in blood cultures; (ii) a differential time to positivity ≥ 2 h between catheter-drawn and peripheral blood cultures obtained simultaneously (within 1 h); (iii) local signs of infection at the catheter insertion site (inflammation and/or purulent discharge); and (iv) absence of an alternative plausible source of infection.

Metastatic infectious complications included infective endocarditis, bone and joint infections, embolic events, and vascular graft or endograft infections. Infective endocarditis was diagnosed by the Endocarditis Team (2018–2024) or by expert clinicians (2015–2017), based on the 2023 International Society for Cardiovascular Infectious Diseases Duke criteria [35]. Septic deep vein thrombosis at the site of the inserted venous catheter was diagnosed by compression/duplex ultrasonography or CT scan showing thrombosis of a deep vein, with or without surrounding inflammation, within 144 h of the first positive blood culture. Persistent bacteremia was defined as recovery of the same pathogen from at least one follow-up blood culture obtained ≥48 h after the index blood culture, and was assessed at 144 h from the first positive blood culture [36].

Statistical analyses were performed using SPSS version 26.0 (SPSS Inc., Chicago, IL, USA). Continuous variables were compared using the Mann–Whitney *U* test, and categorical variables using the chi-square test or Fisher’s exact test, as appropriate. Univariate logistic regression was used to identify factors associated with metastatic infectious complications. Candidate variables for inclusion in the multivariable logistic regression models were selected based on clinical relevance and univariate association with metastatic infectious complications (*p* < 0.1), after excluding collinear covariates (variance inflation factor). Model performance was evaluated using the Hosmer–Lemeshow goodness-of-fit test and the area under the receiver operating characteristic curve. All statistical tests were two-tailed, and a *p* < 0.05 was considered statistically significant.

## 5. Conclusions

In conclusion, the risk of metastatic infectious complications among episodes of CRBSI due to Gram-positive cocci and *Candida* spp. was clinically relevant, with *S. aureus* CRBSI associated with the highest risk. These complications should be actively considered in patients with community-onset infection or persistently positive follow-up blood cultures. Further studies are needed to better characterize the risk of metastatic complications across a broader spectrum of pathogens, including those traditionally associated with lower risk, such as Gram-negative bacteria.

## Figures and Tables

**Table 1:** Comparison of episodes with or without metastatic infectious complications among 1023 episodes with catheter-related bloodstream infections.

	No Metastatic Complications (n = 930)	Metastatic Complications (n = 93)	*p*	OR (95% CI)
Demographics				
Male sex	617 (66)	52 (56)	0.044	0.64 (0.42–0.99)
Age (years)	65 (52–75)	68 (58–77)	0.064	
Comorbidities				
Diabetes mellitus	226 (24)	27 (29)	0.315	1.27 (0.80–2.04)
Chronic kidney disease	210 (23)	30 (32)	0.040	1.63 (1.03–2.59)
Hemodialysis	101 (11)	17 (18)	0.040	1.4 (1.04–3.23)
Malignancy (hematological or solid organ)	392 (42)	36 (39)	0.582	0.87 (0.56–1.34)
Immunosuppression	327 (35)	34 (37)	0.820	1.06 (0.68–1.66)
Cardiac predisposing factors				
Injection drug use	42 (5)	1 (1)	0.170	0.23 (0.31–1.69)
Congenital disease	13 (1)	2 (3)	0.639	1.55 (0.34–6.98)
Prosthetic valve	54 (6)	7 (8)	0.490	1.32 (0.58–2.99)
Moderate or severe valve regurgitation/stenosis	79 (9)	13 (14)	0.087	1.75 (0.93–3.29)
Prior endocarditis	20 (2)	1 (1)	0.713	0.50 (0.07–3.73)
Cardiac implantable electronic devices	52 (6)	13 (14)	0.005	2.74 (1.43–5.25)
Extracardiac prosthetic material				
Endovascular graft	45 (5)	6 (7)	0.454	1.36 (0.56–3.27)
Arthroplasty	72 (8)	10 (11)	0.315	1.44 (0.71–2.89)
Bone-implanted material	40 (4)	2 (2)	0.421	0.49 (0.12–2.06)
Venous catheter				
Peripheral venous catheter	282 (30)	32 (34)	0.411	1.21 (0.77–1.89)
Central venous catheter	710 (76)	67 (72)	0.373	0.80 (0.50–1.29)
Short-term central venous line	427 (46)	29 (31)	0.006	0.53 (0.34–0.84)
Totally implantable venous access device	172 (19)	23 (25)	0.165	1.45 (0.88–2.39)
Peripherally inserted central catheter	134 (14)	12 (13)	0.876	0.88 (0.47–1.66)
Permanent nephrological catheter	44 (5)	13 (14)	0.001	3.27 (1.69–6.33)
Microbiological data				
Isolated pathogen				
*S. aureus*	284 (31)	47 (51)	<0.001	2.32 (1.51–3.57)
Coagulase-negative staphylococci	289 (31)	18 (19)	0.018	0.53 (0.31–0.91)
*Streptococcus* spp.	90 (10)	8 (9)	0.855	0.88 (0.41–1.87)
*Enterococcus* spp.	165 (18)	10 (11)	0.111	0.56 (0.28–1.10)
*Candida* spp.	155 (17)	16 (17)	0.884	1.04 (0.59–1.83)
Persistent bloodstream infection ≥48 h	111 (12)	42 (45)	<0.001	6.08 (3.86–9.57)
Polymicrobial bloodstream infection	142 (15)	14 (15)	1.000	0.98 (0.54–1.78)
Infection data				
Community-onset	105 (11)	26 (28)	<0.001	3.05 (1.86–5.01)
Fever (temperature > 38 °C)	785 (84)	77 (83)	0.656	0.89 (0.50–1.57)
Sepsis or septic shock	365 (39)	45 (48)	0.096	1.45 (0.95–2.23)
Cardiac imaging				
TTE	487 (52)	76 (82)	<0.001	4.07 (2.37–6.99)
TEE	116 (13)	42 (45)	<0.001	5.78 (3.68–9.08)
[^18^F]FDG-PET/CT	57 (6)	26 (28)	<0.001	5.94 (3.51–10.1)
Cardiac CT	10 (1)	4 (4)	0.031	4.14 (1.27–13.5)
Extracardiac imaging				
Thoracoabdominal imaging	419 (45)	75 (81)	<0.001	5.08 (2.99–8.64)
Spinal MRI	38 (4)	7 (8)	0.177	1.91 (0.83–4.41)
Cerebral imaging	165 (18)	39 (42)	<0.001	3.35 (2.15–5.23)
Venous duplex ultrasonography	416 (45)	58 (62)	0.001	2.05 (1.32–3.18)
Catheter-related infection data				
Type of infected catheter			0.535	0.87 (0.54–1.40)
Peripheral venous catheter	234 (25)	26 (28)		
Central venous catheter	696 (75)	67 (72)		
Type of infected central venous catheter				
Short-term central venous line	408 (44)	27 (29)		
Totally implantable venous access device	125 (13)	18 (19)		
Peripherally inserted central catheter	134 (14)	12 (13)		
Permanent nephrological catheter	29 (3)	10 (11)	0.002	3.74 (1.76–7.95)
Catheter removal within 48 h	629 (68)	59 (63)	0.419	0.83 (0.53–1.29)
Septic deep vein thrombosis	119 (13)	24 (26)	0.001	2.37 (1.43–3.92)
Positive tip culture for the same microorganism	300 (32)	38 (41)	0.105	1.45 (0.94–2.24)
Local signs of inflammation	286 (31)	30 (33)	0.814	1.07 (0.68–1.69)
Positive differential time to positivity	140 (15)	17 (18)	0.450	1.26 (0.72–2.20)
Duration of antimicrobial treatment (days)	14 (8–17)	31 (20–44)	<0.001	

Data appear as numbers (%) or median (interquartile range). [^18^F]FDG PET/CT: [^18^F]fluorodeoxyglucose positron emission tomography/CT; CI: confidence interval; OR: odds ratio; TEE: transesophageal echocardiography; TTE: transthoracic echocardiography.

**Table 2:** Comparison of episodes with or without metastatic infectious complications among 331 episodes with catheter-related *S. aureus* bacteremia.

	No Metastatic Infection (n = 284)	Metastatic Infection (n = 47)	*p*	OR (95% CI)
Demographics				
Male sex	183 (64)	30 (64)	1.000	0.97 (0.51–1.85)
Age (years)	68 (56–79)	70 (57–78)	0.633	
Comorbidities				
Diabetes mellitus	76 (27)	13 (28)	0.861	1.05 (0.52–2.09)
Chronic kidney disease	88 (31)	17 (36)	0.501	1.26 (0.66–2.05)
Hemodialysis	29 (10)	9 (19)	0.085	2.0 (0.92–4.74)
Malignancy (hematological or solid organ)	109 (38)	16 (34)	0.628	0.83 (0.44–1.59)
Immunosuppression	91 (32)	15 (32)	1.000	0.99 (0.51–1.93)
Cardiac predisposing factors				
Injection drug use	6 (2)	1 (2)	1.000	1.01 (0.12–8.56)
Congenital disease	5 (2)	2 (4)	0.260	0.25 (0.47–13,2)
Prosthetic valve	11 (4)	4 (9)	0.244	2.31 (0.70–7.58)
Moderate or severe valve regurgitation/stenosis	41 (14)	8 (17)	0.658	1.22 (0.53–2.79)
Prior endocarditis	0 (0)	1 (2)	0.142	
Cardiac implantable electronic devices	20 (7)	7 (15)	0.083	2.31 (0.92–5.81)
Extracardiac prosthetic material				
Endovascular graft	9 (3)	3 (6)	0.389	2.08 (0.54–8.00)
Arthroplasty	30 (11)	7 (15)	0.451	1.48 (0.61–3.60)
Bone-implanted material	12 (4)	1 (2)	0.702	0.49 (0.06–3.88)
Venous catheter				
Peripheral venous catheter	152 (54)	23 (49)	0.637	0.83 (0.45–1.54)
Central venous catheter	149 (53)	25 (53)	1.000	1.03 (0.56–1.91)
Short-term central venous line	75 (26)	7 (15)	0.102	0.49 (0.21–1.14)
Totally implantable venous access device	53 (19)	10 (21)	0.689	1.18 (0.55–2.52)
Peripherally inserted central catheter	31 (11)	5 (11)	1.000	0.97 (0.36–2.64)
Permanent nephrological catheter	13 (5)	7 (15)	0.014	3.65 (1.37–9.69)
Microbiological data				
Persistent bloodstream infection ≥ 48 h	48 (17)	28 (60)	<0.001	7.25 (3.75–14.0)
Polymicrobial bloodstream infection	28 (10)	4 (9)	1.000	0.85 (0.38–3.55)
Infection data				
Community-onset	36 (13)	13 (28)	0.013	2.63 (1.27–5.46)
Fever (temperature > 38 °C)	260 (92)	43 (92)	1.000	0.99 (0.33–3.00)
Sepsis or septic shock	116 (41)	27 (57)	0.039	1.96 (1.05–3.65)
Cardiac imaging				
TTE	212 (75)	41 (87)	0.065	2.32 (0.95–5.69)
TEE	59 (21)	25 (53)	<0.001	4.33 (2.28–8.22)
[^18^F]FDG-PET/CT	25 (9)	15 (32)	<0.001	4.86 (2.32–10.2)
Cardiac CT	6 (2)	2 (4)	0.317	2.06 (0.40–10.5)
Extracardiac imaging				
Thoracoabdominal imaging	106 (37)	39 (83)	<0.001	8.19 (3.69–18.2)
Spinal MRI	11 (4)	4 (9)	0.244	2.31 (0.70–7.58)
Cerebral imaging	38 (13)	24 (51)	<0.001	6.76 (3.47–13.2)
Venous duplex ultrasonography	151 (53)	33 (70)	0.089	2.08 (1.07–4.05)
Catheter-related infection data				
Type of infected catheter			0.755	1.12 (0.60–2.08)
Peripheral venous catheter	141 (50)	22 (47)		
Central venous catheter	143 (50)	25 (53)		
Type of infected central venous catheter				
Short-term central venous line	67 (24)	7 (15)		
Totally implantable venous access device	35 (12)	7 (15)		
Peripherally inserted central catheter	31 (11)	5 (11)		
Permanent nephrological catheter	10 (4)	6 (13)	0.016	4.01 (1.38–11.6)
Catheter removal within 48 h	235 (83)	36 (78)	0.311	0.68 (0.33–1.43)
Septic deep vein thrombosis	62 (22)	19 (40)	0.010	2.43 (1.27–4.64)
Positive tip culture for the same microorganism	112 (39)	20 (33)	0.748	1.14 (0.61–2.13)
Local signs of inflammation	157 (55)	27 (57)	0.874	1.09 (0.59–2.04)
Positive differential time to positivity	21 (7)	6 (13)	0.245	1.83 (0.70–4.81)
Duration of antimicrobial treatment (days)	16 (14–28)	29 (24–44)	<0.001	

Data appear as numbers (%) or median (interquartile range). [^18^F]FDG PET/CT: [^18^F]fluorodeoxyglucose positron emission tomography/CT; CI: confidence interval; OR: odds ratio; TEE: transesophageal echocardiography; TTE: transthoracic echocardiography.

## Data Availability

The data that support the findings of this study are available from the corresponding author upon reasonable request.

## Supplementary Materials

The following supporting information can be downloaded at https://www.mdpi.com/article/10.3390/antibiotics15101015/s1, Table S1: Diagnoses of episodes with recurrent bacteremia caused by the same species within 120 days of the initial episode; Table S2: Univariable and multivariable logistic regression of metastatic infectious complications among 1023 episodes with catheter-related bacteremia; Table S3: Univariable and multivariable logistic regression of metastatic infectious complications among 331 episodes with catheter-related *S. aureus* bacteremia; Table S4: Definition of confirmed bacteremia and contamination.

## Abbreviations

The following abbreviations are used in this manuscript:

[^18^F]FDG-PET/CT	[^18^F]fluorodeoxyglucose positron emission tomography/CT
aOR	Adjusted odds ratio
CHUV	Lausanne University Hospital
CI	Confidence interval
CRBSI	Catheter-related bloodstream infection
ID	Infectious diseases
MALDI-TOF MS	Matrix-assisted laser desorption–ionization time of flight mass spectrometry
