# Supplementary material for: Risk Factors for Metastatic Infectious Complications in Patients with Catheter-Related Bloodstream Infection
Source: Antibiotics (Basel). 2026 Oct 13;15(10):1015. doi: 10.3390/antibiotics15101015 (PMC13700563; doi:10.3390/antibiotics15101015)
Supplement: Supplementary file 1 [file antibiotics-15-01015-s001.zip › antibiotics-4571880-supplementary.pdf]

Table S1. Diagnoses of episodes with recurrent bacteremia caused by the same species within 120 days of the initial episode

	**Species**	**Diagnosis of the initial episode**	**Diagnosis of the subsequent episode**
1	*S. aureus*	Catheter-related[a]	Catheter-related
2	*S. aureus*	Catheter-related[a]	Catheter-related
3	*S. aureus*	Catheter-related[a]	Catheter-related
4	*S. aureus*	Catheter-related[a]	Catheter-related
5	*S. epidermidis*	Catheter-related[a]	Catheter-related
6	*S. epidermidis*	Catheter-related[a]	Catheter-related
7	*S. epidermidis*	Catheter-related[a]	Catheter-related
8	*S. epidermidis*	Catheter-related[a]	Catheter-related
9	*S. epidermidis*	Catheter-related[a]	Catheter-related
10	*S. epidermidis*	Catheter-related[a]	Catheter-related
11	*S. epidermidis*	Catheter-related[a]	Catheter-related
12	*S. epidermidis*	Catheter-related[a]	Catheter-related
13	*S. epidermidis*	Catheter-related[a]	Catheter-related
14	*S. hominis*	Catheter-related[a]	Catheter-related
15	*S. mitis*	Catheter-related[a]	Catheter-related
16	*E. faecalis*	Catheter-related[a]	Catheter-related
17	*E. faecium*	Catheter-related[a]	Catheter-related

18	*E. faecium*	Catheter-related	Catheter-related
19	*E. faecium*	Catheter-related[a,b]	Septic thrombosis of portal vein
20	*C. albicans*	Catheter-related[a]	Catheter-related
21	*C. glabrata*	Catheter-related[a]	Catheter-related
22	*C. dubliniensis*	Catheter-related[a]	Catheter-related

[a]Infected catheter was extracted

[b]Episode with deep vein thrombosis related to central venous catheter and septic pulmonary emboli

Table S2. Univariable and multivariable logistic regression of metastatic infectious complications among 1023 episodes with catheter-related bacteremia

	Univariable analysis		Multivariable logistic regression[a]	
	P	**OR (95% CI)**	***P***	**aOR (95% CI)**
Female sex	0.044	1.56 (1.01-2.39)	0.042	1.61 (1.02-2.56)
Permanent nephrological catheter	0.001	3.26 (1.69-6.33)	0.177	1.72 (0.78-3.78)
S. aureus	<0.001	2.32 (1.51-3.57)	0.048	1.62 (1.01-2.61)
Persistent bloodstream infection ≥48 hours	<0.001	6.08 (3.86-9.57)	<0.001	4.54 (2.78-7.44)
Community-onset	<0.001	3.05 (1.86-5.01)	0.006	2.23 (1.26-3.95)
Sepsis or septic shock	0.096	1.45 (0.95-2.23)	0.152	1.51 (0.88-2.27)
Cardiac implantable electronic devices	0.005	2.74 (1.43-5.25)	0.281	1.38 (0.77-2.46)
Septic deep vein thrombosis	0.001	2.37 (1.43-3.92)	0.038	2.19 (1.04-4.58)

aOR: adjusted odds ratio; CI: confidence interval

[a]Model fit assessed using the Hosmer-Lemeshow goodness-of-fit test. The test was non-significant, chi-square(7)= 10.044, *P*= 0.186, indicating adequate model fit. The model demonstrated moderate discriminatory ability, with an area under the receiver operating characteristic curve (AUROC) of 0.74 (95% CI: 0.68-0.79).

Table S3. Univariable and multivariable logistic regression of metastatic infectious complications among 331 episodes with catheter-related *S. aureus* bacteremia

	**Univariable analysis**		**Multivariable logistic regression**[a]	
	P	**OR (95% CI)**	***P***	**aOR (95% CI)**
Permanent nephrological catheter	0.014	3.65 (1.37-9.69)	0.289	2.07 (0.54-7.94)
Persistent bacteremia ≥48 hours	<0.001	7.25 (3.74-14.0)	<0.001	6.28 (3.12-12.6)
Community-onset	0.013	2.63 (1.27-5.46)	0.507	1.37 (0.54-3.49)
Sepsis or septic shock	0.039	1.96 (1.05-3.65)	0.054	1.96 (0.99-3.88)
Septic deep vein thrombosis	0.010	2.43 (1.27-4.64)	0.383	1.38 (0.67-2.87)

aOR: adjusted odds ratio; CI: confidence interval

[a]Model fit assessed using the Hosmer-Lemeshow goodness-of-fit test. The test was non-significant, chi-square(4)=6.50, *P*=0.164, indicating adequate model fit. The model demonstrated moderate discriminatory ability, with an area under the receiver operating characteristic curve (AUROC) of 0.76 (95% CI: 0.68-0.84).

Table S4. Definition of confirmed bacteremia and contamination

Context[a]	Diagnosis
Episode with at least one positive blood culture set for *Staphylococcus aureus*, *S. lugdunensis, pneumoniae, S. pyogenes, S. agalactiae, or S. dysgalactiae, and Candida* spp.	Confirmed bacteremia
Episode with two positive blood culture sets for enterococcal or viridans streptococcal species	Confirmed bacteremia
Episode with one positive blood culture sets for enterococcal or viridans streptococcal species	
• Received antimicrobial treatment	Confirmed bacteremia
• Resolution of clinical symptoms without antibiotics or source control interventions	Contamination
CoNS was not considered the likely pathogen based on the clinical context (e.g., community-acquired pneumonia)	Contamination
Episode with one positive blood culture set for a CoNS other than *S. lugdunensis*	Contamination
Episode with two positive blood culture sets for a CoNS other than *S. lugdunensis* with different antimicrobial susceptibility profiles	Contamination
Episode with two positive blood culture sets for a CoNS other than *S. lugdunensis* with identical antimicrobial susceptibility profiles, and:	
• CoNS was considered the likely pathogen based on the clinical context (e.g., catheter-related infection) and targeted treatment for CoNS was prescribed	Confirmed bacteremia
• CoNS was not considered the likely pathogen based on the clinical context (e.g., community-acquired pneumonia)	Contamination

[a]all episodes presented clinical signs of infection

CoNS: coagulase-negative staphylococci
